# Contact probing of stretched membranes and adhesive interactions: graphene and other two-dimensional materials

**DOI:** 10.1098/rspa.2016.0550

**Published:** 2016-11

**Authors:** Feodor M. Borodich, Boris A. Galanov

**Affiliations:** 1 School of Engineering, Cardiff University, Cardiff CF24 0AA, UK; 2 Institute for Problems in Materials Science, National Academy of Sciences of Ukraine, 3 Krzhyzhanovsky Street, Kiev 03142, Ukraine

**Keywords:** two-dimensional materials, graphene, membrane, Johnson, Kendall and Roberts theory, adhesion, nanoindentation

## Abstract

Contact probing is the preferable method for studying mechanical properties of thin two-dimensional (2D) materials. These studies are based on analysis of experimental force–displacement curves obtained by loading of a stretched membrane by a probe of an atomic force microscope or a nanoindenter. Both non-adhesive and adhesive contact interactions between such a probe and a 2D membrane are studied. As an example of the 2D materials, we consider a graphene crystal monolayer whose discrete structure is modelled as a 2D isotropic elastic membrane. Initially, for contact between a punch and the stretched circular membrane, we formulate and solve problems that are analogies to the Hertz-type and Boussinesq frictionless contact problems. A general statement for the slope of the force–displacement curve is formulated and proved. Then analogies to the JKR (Johnson, Kendall and Roberts) and the Boussinesq–Kendall contact problems in the presence of adhesive interactions are formulated. General nonlinear relations among the actual force, displacements and contact radius between a sticky membrane and an arbitrary axisymmetric indenter are derived. The dimensionless form of the equations for power-law shaped indenters has been analysed, and the explicit expressions are derived for the values of the pull-off force and corresponding critical contact radius.

## 1. Introduction

For many years, it was assumed that atomically thin two-dimensional (2D) materials could not exist in the free state due to their instability (e.g. a discussion by Meyer *et al.* [1]). The group led by Geim showed that layered materials may be exfoliated using a sticky tape; in particular, one can produce atomically thin 2D carbon allotrope, named graphene, and that graphene is stable [1,2]. The group led by Ruoff showed that there are other methods for producing graphene-based materials (like graphene oxide) in significant quantities [3,4]. Currently, 2D materials are a wide new field in condensed matter research [5]. In addition to graphene and graphene oxide these 2D materials include mica, weakly coupled planes of *CuO*_2_, *MoS*_2_, *NbSe*_2_ *WSe*_2_, *TaS*_2_, *TaSe*_2_, etc. [5–7]. These 2D materials have a great potential for applications in modern electronics. Indeed, it is known that graphene has remarkable electronic properties, high tensile strength and low density (e.g. [8,9]), hence it is considered as an excellent material for nanoelectromechanical systems (NEMSs). Examples of these systems include the circular drum resonators covered by graphene, pressure sensors and membrane-based NEMS devices with suspended graphene spanned between electrodes (e.g. [10–12]).

Nowadays studies of 2D materials are concentrated not only on their remarkable electronic properties but also on their mechanical behaviour. Contact probing is the preferable method for studying mechanical properties of thin 2D materials (e.g. [4,13–15]). A comprehensive review of 2D materials, methods of their fabrications and studies of their mechanical properties have been recently published by Castellanos-Gomez *et al.* [7]. Usually, the probe of an atomic force microscope (AFM) or a nanoindenter is used. If a probe is applied at the centre of a suspended 2D material, i.e. at the centre of a circular membrane or in the middle of a doubly clamped beam, then one can record the external load (*P*) and the corresponding displacement (*δ*) of the indenter in order to get the force–displacement curve. This is the so-called depth-sensing nanoindentation. Using analysis of the *P*–*δ* curve, one can try to extract the mechanical properties of the material. Usually, the classic *P*–*δ* expressions [16] for doubly clamped structures have been used [7]. However, these expressions are valid for structures of finite thickness of non-zero bending rigidity. There is a need for studying contact problems for atomically thin 2D materials.

In nanoindentation tests of 2D materials, deformations of a freestanding membrane may depend not only on the force *P* acting on the probe but also on the attraction between the probe tip and the material due to interaction forces having electromagnetic origin. Discussing the phenomenon of adhesion, Robert Hooke wrote ‘for the Congruity, in the Vibrative motions, may be the cause of all kind of attraction, not only Electrical, but Magnetical also, and therefore it may be also of Tenacity and Glutinousness.’ [17]. Peter Lebedev [18] gave the first electromagnetic explanation for the nature of the van der Waals (vdW) forces. Later it was realized that the vdW forces include forces of different origins (the Keesom, Debye and London forces) that mean, respectively, attraction between: two permanent dipoles, a permanent dipole and a corresponding induced dipole and two instantaneously induced dipoles [19,20]. Various ways for description of vdW interactions and adhesion between solids are still discussed by the scientific community. Recently, it has been argued that a qualitatively correct description of the vdW interactions between polarizable nanostructures over a wide range of finite distances can only be attained by accounting for the wavelike nature of charge density fluctuations [21]. Molecular adhesion becomes increasingly significant as the contact size decreases [20], hence, one can expect that adhesive interactions may have a great influence on the contact between a probe and a sample of a 2D material.

There are various approaches to adhesive contact problems. In particular, these approaches may include the use of the Derjaguin approximation and the energy approach [22] and the use of an appropriate interaction potential between points on the surfaces, for example, a Lennard–Jones potential or piecewise-constant approximations of these potentials [23,24]. Nowadays, there are several classic approaches to describe adhesive contact between solid spheres that include the JKR (Johnson, Kendall and Roberts) and the DMT (Derjaguin–Muller–Toporov) theories that may be considered as limits of the Maugis transition between the JKR and DMT approaches [23,25,26]. To study contact problems with molecular adhesion one needs to know the work of adhesion, *w* that is equal to the energy needed to separate two dissimilar surfaces from contact to infinity. The above classic models are very helpful for studying various phenomena that involve molecular adhesion. For example, the non-direct BG method for the experimental determination of the work of adhesion and elastic contact modulus of materials [27] is based on the use of these classic models. It has been shown recently that this non-direct method is fast and robust [28]. The similar techniques should be developed for problems of adhesive contact between an indenter and a stretched membrane of 2D material.

Thus, for proper understanding of interactions between the tip and a 2D membrane, one needs to study the influence of the adhesive forces on their contact. Graphene may be considered as one of the main classes of 2D materials. Although adhesive contact problems for elastic solids and elastic membranes have been studied for a long period of time (e.g. [29,30] and reference therein) and the contact probing of graphene membranes is a very popular technique (e.g. [7]) to the best of our knowledge, the adhesive contact problem for atomically thin object (e.g. graphene layer) has not been studied yet. In addition, the used theoretical models assumed non-zero bending rigidity of the membranes. In this paper, a 2D layer under tensile loading is modelled as a linear elastic membrane whose bending rigidity may be neglected. The membrane is isotropic because it is known [31] that the elastic in-plane properties of 2D graphene layer in a linear approximation are isotropic. To solve contact problems with molecular adhesion for a stretched membrane, the JKR approach along with Derjaguin’s ideas will be employed. The former approach involves calculations of the work done by the surface attractions and the work of deformation in the elastic objects [22,25].

The paper is organized as follows:

In §2, we present some preliminary information about graphene and some other carbon-based materials. Then we discuss formulations of the classic frictionless non-adhesive Hertz and Boussinesq contact problems and mechanics of adhesive contact for elastic three-dimensional (3D) solids.

In §3, we first discuss equations of elastic stretched membranes. Then for the stretched 2D membranes, we formulate the non-adhesive frictionless contact problems that are analogies to the corresponding Hertz and Boussinesq contact problems. These new problems are solved using the Green function approach. For the problems under consideration, a general statement about slopes of the force–displacement relations is formulated and proved.

The JKR theory of adhesive contact was originally developed for linearly elastic isotropic spheres. In §4, the adhesive contact between a stretched graphene membrane and an axisymmetric convex punch of arbitrary profile is formulated and solved in the framework of the JKR theory. The derivation of the main relations is quite straightforward because it is based on the use of the above mentioned general statement. Then connections between the obtained results and problems of adhesive probing of graphenes are discussed assuming that the indenter shape is described by a power-law function of an arbitrary real degree *m*>1. In this case, the exact relations between the actual force, the indenter displacements and contact radius have been derived and the explicit expressions are found for the values of the pull-off force and for the corresponding critical contact radius. Special attention is given to problems for spherical and flat-ended circular punches.

## 2. Preliminaries

We use both the Cartesian and cylindrical coordinate frames, namely *x*_1_=*x*,*x*_2_=*y*,*x*_3_=*z* and *r*,*φ*,*z*, where $r=\sqrt{x^{2}+y^{2}}$ and x=rcos⁡φ, y=rsin⁡φ.

### (a) Graphene and other carbon-based materials

Graphene may be considered as the main example of 2D materials. The term graphene denotes a carbon allotrope that can be described as an atom thick sheet made of its atoms. In fact, graphene is a single layer of graphite, i.e. the atoms of graphene are densely packed into a 2D honeycomb crystal lattice. Owing to symmetry of the graphene lattice, a hexagonal ring may be used to represent its structure. The initial length of the hexagon side (carbon interatomic distance) is *a*=1.42 Å and the initial angle between two any sides is *α*=120^°^. Properties of carbon allotropes were studied for many years (e.g. reviews by Derjaguin & Fedoseev [32]). Initially, these studies were concentrated on graphite and diamond allotropes. Tubular carbon allotropes that later started to be called carbon nanotubes (CNTs) were discovered only in 1950 due to the electron microscopy studies fulfilled by Radushkevich & Lukyanovich [33,34] in the Institute of Physical Chemistry (USSR Academy of Sciences) where an extended programme on carbon research was led by Derjaguin (Department of Surface Forces) and Dubinin (Department of Sorption Processes). The results of diffraction studies of the carbon hollow whiskers (CNTs) showed that CNTs are made from the pure graphene planes elongated along the tubes and this can explain high tensile strength of the CNTs [35]. Thus, graphene is a basic building block for many graphitic materials of other dimensionalities [36]. The mechanical properties of graphene have been experimentally studied using various approaches (e.g. [10,37,38]). On the theoretical front the elastic in-plane properties of graphene were studied using various linearized discrete models and models involving various nonlinear multi-body potentials (e.g. discussions in [39,40]).

The carbon atoms in the graphene crystal are bonded to each other by covalent bonds. It is clear that a linearization of interatomic interactions will represent the covalent bonds as an elastic spring whose elastic constant is one of the discrete model parameters. A comprehensive review of existing approaches to modelling the elastic in-plane properties of graphene has been presented by Berinskii & Borodich [41]. It was shown that a number of popular linear and nonlinear models of graphene lattices (in particular, popular nonlinear base on the use of the Tersoff or Brenner potentials) have serious flaws and often the results obtained using these models do not have physical meaning. On the other hand, the two-parametric molecular mechanics model developed by Gillis [42] where parameters represent the central and non-central interaction of carbon atoms, can well describe the experimental results for small in-plane deformations of graphene. Nowadays, it is still not clear what is better to consider as thickness of the graphene layer and to avoid this problem, the elastic modulus for graphene and nanotubes are often defined as a product of the conventional Young’s modulus with the layer thickness (see a discussion in [41]). The rigorous studies of the graphene crystal lattice as 2D linear elastic continuum [31] showed that the lattice has isotropic elastic properties.

The graphene oxide (G–O) is another 2D carbon-based material. It is a graphene plane contaminated by sp3-hybridized carbons bearing hydroxyl and epoxide functional groups on the plane, and by carboxyl and carbonyl groups at the plane edges. As it was noted by Suk *et al.* [4], the detailed understanding of G–O structure is still being developed. One may assume that the main arguments presented by Berinskii & Borodich [31] in application to pure graphene, are also valid in application to G–O and therefore, this 2D material has also isotropic elastic properties.

### (b) The Hertz and Boussinesq contact problems for an elastic-half-space

The non-adhesive 3D Hertz formulation assumes that initially there is only one point of contact between two elastic solids. In a geometrically linear formulation of the boundary-value contact problem, each solid is modelled as a positive half-space *x*_3_≥0. The boundary plane *x*_3_=0 is denoted by $R^{2}$. The origin (*O*) of Cartesian *x*_1_,*x*_2_,*x*_3_ coordinates is at the point of initial contact between the solids. Assuming that contact is frictionless and that the radial displacements may be neglected, Hertz [43] gave a formulation of the boundary-value problem (see for details [44]). In particular, the Hertz type of contact problems means that the contact region is unknown in advance, and only vertical displacements of the boundary are taken into account. Hence, one needs to find the finite region *D* of the points at which the bodies are in mutual contact, the relative approach of the bodies *δ*>0 and the displacements and the stresses within the solids.

It is possible to show that the problem formulation is mathematically equivalent to the problem of contact between a positive half-space and a rigid indenter (punch) whose shape function *f* is equal to the initial distance between the surfaces. Hence, the equation of the punch surface given by a function *f*, can be written as *x*_3_=−*f*(*x*_1_,*x*_2_), *f*≥0. After the punch contacts with the half–space, displacements *u*_*i*_ and stresses *σ*_*ij*_ are generated.

Using a known expression for a potential of an ellipsoid, the contact problem for solids whose shapes are approximated as elliptic paraboloids, was solved by Hertz [43]. Hertz showed also that the contact region of the problem is an ellipse. Boussinesq [45] presented independently solutions to other contact problems. The Boussinesq contact problem for a flat-ended cylinder assumes that the contact region is always a circle of a fixed radius.

### (c) The adhesive contact models

Derjaguin [22] presented the first attempt to consider the problem of adhesion between elastic spheres or between an elastic sphere and an elastic half-space. He assumed that the deformed shape of the sphere can be calculated by solving the Hertz contact problem (this assumption was not correct) and suggested to calculate the adhesive interaction using the so-called Derjaguin approximation (this approximation is very useful). This approximation does not employ the pairwise summation of the interactions between all elements of solids, but it reduces the volume molecular attractions to surface interactions. The Derjaguin idea that the virtual work done by the external load is equal to the sum of the virtual change of the potential elastic energy and the virtual work that will be consumed by the increase of the surface attractions (see (21) in Derjaguin [22]), is the cornerstone of many approaches to adhesive contact problems. For example, Sperling [46] used this idea to calculate the full energy of the system and derived the equations that are identical to equations of the JKR theory (see a discussion in [44] for details). However, the JKR approach is more elegant. Indeed, to get the final result, Sperling [46] solved the problem using rather complicated calculations, while according to the JKR approach, the problem may be solved by assuming that the contact system has come to its real state in two steps: (i) first it has got real (true) contact radius *a*_1_ and an apparent depth of indentation *δ*_1_ under some apparent Hertz load *P*_1_, then (ii) using the Boussinesq solution for contact a flat-ended punch of radius *a*_1_, the system is unloaded from *P*_1_ to a real value of the external load *P*_0_ (the true external load) keeping the contact radius *a*_1_ constant. The original JKR approach has the same assumptions as the frictionless axisymmetric Hertz contact. Although the approach was applied to a sphere described as a paraboloid of revolution *z*=*r*^2^/(2*R*), very far generalizations of the approach are possible. Recently, the approach has been extended to non-slipping boundary conditions [47], transversely isotropic solids [48] and the punches of arbitrary axisymmetric blunt shapes contacting elastic materials with rotational symmetry of their elastic properties [44]. It is attempted here to follow the original JKR approach as closely as possible, hence we denote as *a*_1_, *P*_0_ and *δ*_2_ the true values of the radius of adhesive contact, the external load and the displacement, respectively.

The total energy *U*_T_ is obtained by summation of the stored elastic energy *U*_E_, the mechanical energy in the applied load *U*_M_ and the surface energy *U*_S_

2.1 $$U_{T}=U_{E}+U_{M}+U_{S}.$$

According to the Derjaguin approximation and the JKR assumption that the adhesive interactions out of the contact region are negligible, the surface energy can be written as

2.2 $$U_{S}=-wA,$$

where *A* is the area of the contact region.

Sperling [46] argued that the total energy has minimum at equilibrium, whereas Johnson *et al.* [25] argued that the energy satisfies the Griffith criterion. However, both arguments lead to the same equation

2.3 $$\frac{dU_{T}}{da_{1}}=0\;\text{or}\;\frac{dU_{T}}{dP_{1}}=0.$$

It is known that the frictionless JKR results can be also obtained by linear fracture mechanics approach [23]. If *G* is the energy release rate at the edge of the contact then in the frictionless case, the equilibrium is given by *G*=*w* (Griffith’s criterion). However, the concepts of fracture mechanics cannot be directly applied in the case of atomically thin 2D membranes. Thus, the energy approach will be used further.

## 3. Contact problems for a circular graphene membrane: analogies to the Hertz and Boussinesq problems

Owing to specific character of 2D materials, it is reasonable to model a material layer as a membrane whose bending rigidity is negligible. Modelling the material as an elastic membrane allows us to use a geometrically linear formulation of the boundary-value problem like the classic contact problems have. Of course, the formulation has to be slightly modified. We consider only axisymmetric problems of frictionless contact between punches described as bodies of revolution and a circular stretched membrane (a circular drum). Evidently, the above Hertz and Boussinesq contact problems formulated for an elastic-half-space have to be reformulated to reflect the features of a 2D solid.

### (a) The elastic membrane equation and the Green function solution

Mathematical problems related to stretched membranes have been studied by many researchers (e.g. [49,50]). Hence, we provide here just formulations of the appropriate boundary-value problem. Let us consider an elastic membrane *Ω* that is supported by a rigid frame in the horizontal plane $R^{2}$. Let the membrane be held under a uniform tension *T*, i.e. initially the membrane is stretched. Hence, each point (*x*,*y*) of the closure $\bar{\Omega }$ of *Ω* represents a material point of the membrane when it is stretched without any other applied force.

If some external force of density *F*(*x*,*y*) is applied perpendicularly to the membrane surface then vertical displacements of the membrane *u*_3_(*x*,*y*) appear. It is assumed that the internal forces lay in the tangent plane to the deformed surface. Thus, the shape of the deformed surface at equilibrium is presented as (*x*,*y*,*u*_3_(*x*,*y*)) and the tension *T* does not depend on *u*_3_(*x*,*y*), i.e. *T* is constant. The function *u*_3_(*x*,*y*) satisfies the elastic membrane equation that actually is the following Poisson equation

3.1 $$T\nabla^{2}u_{3}(x,y)=-F(x,y),\;(x,y)\in \Omega ,\;\nabla^{2}=\frac{1}{r}\frac{\partial }{\partial r}\left(r\frac{\partial }{\partial r}\right)+\frac{1}{r^{2}}\frac{\partial^{2}}{\partial \varphi^{2}}=\frac{\partial^{2}}{\partial x^{2}}+\frac{\partial^{2}}{\partial y^{2}},$$

where ∇^2^ is the Laplace operator.

If it is assumed additionally that the stretched membrane sticks to the border of the frame ∂*Ω*, then one has a homogeneous Dirichlet boundary condition

3.2 $$u_{3}(x,y)=0,\;(x,y)\in \partial \Omega .$$

Let us consider a circular drum, i.e. a circular membrane that is supported by a rigid circular frame of radius *R*. The Green function *G*(*r*,*φ*,*ρ*,*ψ*) for a circular membrane having the Dirichlet boundary condition can be written as [51]

3.3 $$G(r,\varphi ,\rho ,\psi )=\frac{1}{4\pi }\ln\frac{R^{2}+r^{2}\rho^{2}/R^{2}-2r\rho \cos(\varphi -\psi )}{r^{2}+\rho^{2}-2r\rho \cos(\varphi -\psi )}$$

and the solution *u*_3_(*r*,*φ*) to the elastic membrane equation (3.1) with homogeneous boundary condition (3.2) is presented in the form

3.4 $$u_{3}(r,\varphi )=\frac{1}{T}\int_{\Omega }G(r,\varphi ,\rho ,\psi )F(\rho ,\psi )\rho \;d\rho \;d\psi =\frac{1}{T}\int_{0}^{2\pi }\int_{0}^{R}G(r,\varphi ,\rho ,\psi )F(\rho ,\psi )\rho \;d\rho \;d\psi .$$

In the case of axial symmetry, the membrane deflection is only a function of the radius *r* and it does not depend on the angle *φ*, i.e. *u*_3_(*r*,*φ*)≡*u*_3_(*r*) . Hence, the partial derivative ∂/∂*r* can be replaced by the total derivative d/d*r*. The elastic membrane equation (3.1) for this particular case could be derived in a very simple way (e.g. [49]). Indeed, the vertical component of the tension acting on a sector of the membrane with the central angle Δ*φ* is equal to *Tr*Δ*φ* d*u*_3_/d*r*. Therefore, the equation of equilibrium of the membrane along the vertical axis *z* under a vertical force of intensity *F*(*r*) per unit area is

$$\frac{d}{dr}\left(Tr\Delta \varphi \frac{du_{3}}{dr}\right)+F(r)r\Delta \varphi =0.$$

In this case, the equation (3.1) and the boundary condition (3.2) can be written as

3.5 $$T\frac{1}{r}\frac{d}{dr}\left(r\frac{du_{3}}{dr}\right)+F(r)=0,\;0\leq r\leq R$$

and

3.6 $$u_{3}(r)=0,\;r=R.$$

### (b) An analogy to the Hertz-type contact problem

**Figure 1.**
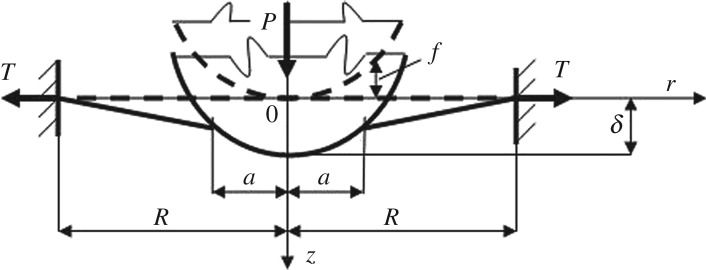
A schematic of contact between a convex smooth axisymmetric punch *f*(*r*) and an elastic membrane. Dash lines correspond to the problem without an external load (*P*=0) and the solid lines describe the system state after application of the load *P*. Here *T* is the tension in the membrane; *R* and *a* are the radii of the drum and the contact region, respectively, and *δ* is the displacement of the punch nose.

If one considers an analogy to the axisymmetric Hertz-type contact problem then for a convex smooth punch *f*(*r*) pressed by a vertical load *P*, one needs to find the radius *a* of unknown region *D*_C_ of contact between the punch and the graphene membrane and the displacement of the punch nose *δ* (figure 1).

It is known that within the contact region the displacements are given by

3.7 $$u_{3}(r)=\delta -f(r),\;r\leq a.$$

In the general case, the contact pressure *p*(*r*) is acting along *z* axis, i.e. it creates positive force density

3.8 $$p(r)=F(r)=-T\frac{1}{r}\frac{d}{dr}\left(r\frac{du_{3}}{dr}\right)=T\frac{1}{r}\frac{d}{dr}\left(r\frac{df(r)}{dr}\right),\;r\leq a$$

because *u*′_3_(*r*)=−*f*′(*r*) for *r*≤*a*; and *p*(*r*)=0 for *r*>*a*. Note the pressure may be negative due to the presence of adhesive interactions.

The total vertical load acting on the membrane *P* satisfies the equation

3.9 $$\int_{0}^{2\pi }\int_{0}^{R}F(r)r\;dr\;d\varphi =2\pi \int_{0}^{R}F(r)r\;dr=P.$$

Hence, for an axisymmetric convex smooth punch *f*(*r*), *f*(0)=0 acting on the membrane, one has

3.10 $$P=2\pi T\int_{0}^{a}\frac{d}{dr}\left(r\frac{df(r)}{dr}\right)dr=2\pi Taf^{\prime }(a).$$

Here we have used the Newton fluxion notation *f*′ to denote a derivative of *f*.

Note that, in the general case of an axisymmetric convex smooth punch *f*(*r*), *f*(0)=0 acting on the membrane, one has *δ*=*u*_3_(0,0). It follows from (3.3) that

$$G(0,0,\rho ,\psi )=\frac{1}{4\pi }\ln\frac{R^{2}}{\rho^{2}}.$$

Substituting the above expression and (3.8) into (3.4), one obtains

3.11 $$\delta =u_{3}(0,0)=\frac{1}{T}\int_{0}^{2\pi }\int_{0}^{a}\frac{1}{4\pi }\ln\frac{R^{2}}{\rho^{2}}\cdot T\frac{1}{\rho }\frac{d}{d\rho }\left(\rho \frac{df(\rho )}{d\rho }\right)\rho \;d\rho \;d\psi$$

or

3.12 $$\delta =\frac{2\pi }{4\pi }\int_{0}^{a}\ln{\left(\frac{R}{\rho }\right)}^{2}\frac{d}{d\rho }\left(\rho \frac{df}{d\rho }\right)d\rho =af^{\prime }(a)\ln\left(\frac{R}{a}\right)+f(a).$$

If the punch shape is described by monomial (power-law) function of radius of an arbitrary real degree *m*≥1

3.13 $$f(r)=B_{m}r^{m},$$

where *m* is the degree of the monomial function and *B*_*m*_ is the constant of the shape, then one has

3.14 $$p(r)=\left\{ \begin{array}{ll} TB_{m}m^{2}r^{m-2} & \text{for}\;r\leq a\\ 0 & \text{for}\;r>a. \end{array}\right.$$

Hence, it follows from (3.10) that

3.15 $$a={\left(\frac{P}{2\pi mTB_{m}}\right)}^{1/m}.$$

For a power-law punch (3.13), it follows from (3.11) that

3.16 $$\delta =B_{m}a^{m}\left[\ln{\left(\frac{R}{a}\right)}^{m}+1\right]=\frac{P}{2\pi mT}\left[1-\ln\left(\frac{P}{2\pi mTB_{m}R^{m}}\right)\right].$$

Let us derive a general relation for slopes of *δ*–*P* curves similar to the relation derived by Borodich [44] in the case of frictionless Hertz-type contact problem. The following general statement for the slope of the force–displacement curve can be formulated and proved.

Let a circular linear elastic isotropic membrane be supported by a rigid circular frame of radius *R* and the membrane be stretched by a uniform tension *T*. Let the behaviour of the membrane be described by (3.5) and (3.6). Let an axisymmetric convex, smooth ($f\in C^{2}(R^{2}\setminus \{0\})$) punch *f*(*r*), *f*(0)=0 be in contact with the membrane under action of the load *P* and the contact problem be described by (3.7)–(3.9).

Then the slope of the *δ*–*P* curve at any point is

3.17 $$\frac{dP}{d\delta }=K,\;K=\frac{2\pi T}{\ln(R/a)},$$

where *a* is the radius of the contact region.

Indeed, it follows from (3.10) that *P*=2*πTaf*′(*a*). Then one has

3.18 $$\frac{dP}{da}=2\pi T[f^{\prime }(a)+af^{\prime \prime }(a)].$$

On the other hand, it follows from (3.12) that $\delta =[af^{\prime }(a)\ln(R/a)+f(a)]$ and, therefore,

3.19 $$\frac{d\delta }{da}=\left[f^{\prime }(a)\ln\left(\frac{R}{a}\right)+af^{\prime \prime }(a)\ln\left(\frac{R}{a}\right)-af^{\prime }(a)\frac{1}{a}+f^{\prime }(a)\right]=\ln\left(\frac{R}{a}\right)[f^{\prime }(a)+af^{\prime \prime }(a)].$$

Comparing (3.18) and (3.19), one obtains

$$\frac{dP}{da}=K\frac{d\delta }{da}$$

that leads to (3.17).

### (c) An analogy to the Boussinesq contact problem

**Figure 2.**
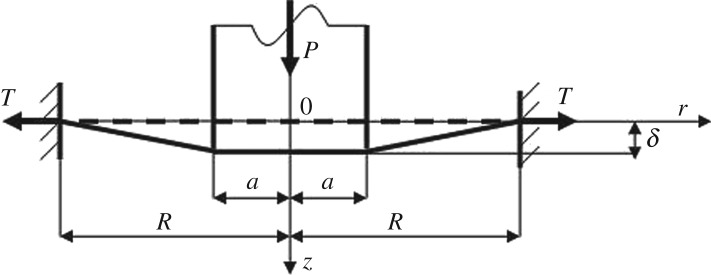
A schematic of contact between a flat-ended axisymmetric punch and an elastic membrane. Dash lines correspond to the position of the membrane without action of an external load (*P*=0) and the solid lines describe the system state after application of the load *P*. Here *T* is the tension in the membrane; *R* and *a* are the radii of the drum and the punch, respectively, and *δ* is the displacement of the punch.

If one considers an analogy to the axisymmetric Boussinesq contact problem for a flat-ended punch of radius *a* pressed by a vertical load *P* (figure 2), then it follows from (3.5) that the pressure *p*(*r*)=0 for 0≤*r*<*a*. Hence, one has

p(r)=Cδ(r−a),

where *δ*(*r*−*a*) is the Dirac delta-function having its support on the circle of radius *a* and the constant *C* is defined from the condition that the total vertical load acting on the membrane *P* satisfies the equation

3.20 $$P=\int_{0}^{2\pi }\int_{0}^{R}p(r)r\;dr\;d\varphi =\int_{0}^{2\pi }\int_{0}^{R}C\delta (r-a)r\;dr\;d\varphi =2\pi aC.$$

Hence, *C*=*P*/(2*πa*).

Owing to axial symmetry of the problem, *u*_3_(*r*,*φ*)=*u*_3_(*r*,0)=*u*_3_(*r*), i.e. one can put *φ*=0 in the expression for the Green function and correspondingly into (3.4)

$$u_{3}(r)=\frac{C}{T}\frac{1}{4\pi }\int_{0}^{2\pi }\int_{0}^{R}\ln\frac{R^{2}+r^{2}\rho^{2}/R^{2}-2r\rho \cos\psi }{r^{2}+\rho^{2}-2r\rho \cos\psi }\delta (\rho -a)\rho \;d\rho \;d\psi$$

or

3.21 $$u_{3}(r)=\frac{Ca}{T}\frac{1}{4\pi }\int_{0}^{2\pi }\ln\frac{R^{2}+r^{2}a^{2}/R^{2}-2ra\cos\psi }{r^{2}+a^{2}-2ra\cos\psi }\;d\psi .$$

Using a known expression (e.g. 865.73 at [52])

3.22 $$\int_{0}^{2\pi }\ln(k^{2}\pm 2kl\cos\psi +l^{2})\;d\psi =4\pi \ln k,\;\text{for}\;0\leq l\leq k,$$

one can calculate the membrane deviation by rearranging the above integral (3.21), into the following form

3.23 $$u_{3}(r)=\frac{Ca}{T}\frac{1}{4\pi }\int_{0}^{2\pi }\ln\frac{R^{2}/(ra)+ra/R^{2}-2\cos\psi }{r/a+a/r-2\cos\psi }\;d\psi .$$

Let us consider the membrane deflection outside the contact region, i.e. the case *a*≤*r*≤*R*. Putting *k*^2^=*R*^2^/(*ra*) and *l*^2^=*ra*/*R*^2^, and applying (3.22) to the nominator of (3.23) and then putting *k*^2^=*r*/*a* and *l*^2^=*a*/*r* and applying (3.22) to the denominator, one gets

3.24 $$u_{3}(r)=\frac{Ca}{T}\frac{1}{4\pi }4\pi \left(\ln\frac{R}{\sqrt{ra}}-\ln\sqrt{\frac{r}{a}}\right)=\frac{aC}{T}\ln\frac{R}{r}=\frac{P}{2\pi T}\ln\frac{R}{r}.$$

Let us consider now the membrane deflection inside the contact region, i.e. the case 0≤*r*≤*a*. Putting *k*^2^=*R*^2^/(*ra*) and *l*^2^=*ra*/*R*^2^, and applying (3.22) to the nominator of (3.21) and then putting *k*^2^=*a*/*r* and *l*^2^=*r*/*a* and applying (3.22) to the denominator, one gets

3.25 $$u_{3}(r)=\frac{Ca}{T}\frac{1}{4\pi }4\pi \left(\ln\frac{R}{\sqrt{ra}}-\ln\sqrt{\frac{a}{r}}\right)=\frac{aC}{T}\ln\frac{R}{a}=\frac{P}{2\pi T}\ln\frac{R}{a}=\mathrm{const}.$$

The logarithmic decay in the membrane solution is in agreement with known solutions that were obtained for membranes of finite thickness (e.g. [29,30]).

Thus, it follows from (3.25) that the displacement *δ* of a flat-ended punch of radius *a* acting on an elastic membrane of radius *R* and stretched by the uniform tension *T* (figure 2), is a linear function of the external load *P*

3.26 $$\delta =u_{3}(r)=\frac{P}{K},\;K=\frac{2\pi T}{\ln(R/a)},\;0\leq r\leq a.$$

The formulae (3.24) and (3.25) give the full solution to the contact problem for a circular flat-ended punch.

## 4. Adhesion to a circular graphene monolayer membrane

Here the JKR theory will be extended to a circular graphene monolayer membrane. The original theory was developed for adhesive contact between two isotropic elastic spheres.

### (a) General expressions for an arbitrary axisymmetric punch

As one neglects the adhesive forces acting outside the contact region, it follows from (2.2) that the surface energy can be written as

4.1 $$U_{S}=-w\pi a_{1}^{2}.$$

According to the JKR formalism, we calculate the elastic energy of the system *U*_E_ as the difference between the elastic energies (*U*_E_)_1_ on loading and (*U*_E_)_2_ on unloading branches. Therefore, the stored elastic energy *U*_E_ is

4.2 $$U_{E}={\left(U_{E}\right)}_{1}-{\left(U_{E}\right)}_{2},$$

where

4.3 $${\left(U_{E}\right)}_{1}=P_{1}\delta_{1}-\int_{0}^{P_{1}}\delta (P)\;dP$$

at the loading of a curved axisymmetric punch until the fictitious load *P*_1_ that corresponds to the true contact radius *a*_1_; and

4.4 $${\left(U_{E}\right)}_{2}=\int_{P_{0}}^{P_{1}}\delta (P)\;dP=\int_{P_{0}}^{P_{1}}\frac{P}{K(a_{1})}\;dP=\frac{P_{1}^{2}-P_{0}^{2}}{2K(a_{1})}$$

for a flat-ended punch of radius *a*_1_ that is unloaded from *P*_1_ to the true contact load *P*_0_. The above solution (3.26) for a flat-ended circular punch has been used to calculate (4.4). Thus

$$U_{E}={\left(U_{E}\right)}_{1}-{\left(U_{E}\right)}_{2}=P_{1}\delta_{1}-\int_{0}^{P_{1}}\delta (P)\;dP-\frac{P_{1}^{2}-P_{0}^{2}}{2K(a_{1})}.$$

The mechanical work of the applied load is calculated as

4.5 $$U_{M}=-P_{0}\delta_{2}=-P_{0}(\delta_{1}-\Delta \delta ),$$

where Δ*δ*=*δ*_1_−*δ*_2_ is the change in the depth of penetration due to unloading. Taking into account the solution (3.26), one obtains

$$\Delta \delta =\frac{P_{1}-P_{0}}{K(a_{1})},$$

and therefore, one has

$$U_{M}=-P_{0}\delta_{2}=-P_{0}\left(\delta_{1}-\frac{P_{1}-P_{0}}{K(a_{1})}\right).$$

The total energy *U*_T_ is calculated according to (2.1). Therefore, one has

$$U_{T}=P_{1}\delta_{1}-\int_{0}^{P_{1}}\delta (P)\;dP-\frac{\left(P_{1}^{2}-P_{0}^{2}\right)}{2K(a_{1})}-P_{0}\delta_{1}+P_{0}\frac{\left(P_{1}-P_{0}\right)}{K(a_{1})}-w\pi a_{1}^{2}.$$

Taking into account that

$$-\frac{\left(P_{1}^{2}-P_{0}^{2}\right)}{2K(a_{1})}+P_{0}\frac{\left(P_{1}-P_{0}\right)}{K(a_{1})}=-\frac{{\left(P_{1}-P_{0}\right)}^{2}}{2K(a_{1})},$$

one has

4.6 $$U_{T}=(P_{1}-P_{0})\delta_{1}-\int_{0}^{P_{1}}\delta (P)\;dP-\frac{{\left(P_{1}-P_{0}\right)}^{2}}{2K(a_{1})}-w\pi a_{1}^{2}.$$

This expression is an analogy to the expression obtained for a generalized JKR contact theory in the case of 3D elasticity [44].

Because the equilibrium state satisfies the equation (2.3), we need to calculate the appropriate derivatives. First, let us get the following expressions

$$\begin{array}{rl} \frac{d}{dP_{1}}[(P_{1}-P_{0})\delta_{1}] & =(P_{1}-P_{0})\frac{d\delta_{1}}{dP_{1}}+\delta_{1},\;\frac{d}{dP_{1}}\int_{0}^{P_{1}}\delta (P)\;dP=\delta (P_{1})=\delta_{1},\\ \frac{d}{dP_{1}}\frac{{\left(P_{1}-P_{0}\right)}^{2}}{2K(a_{1})} & =\frac{\left(P_{1}-P_{0}\right)}{K(a_{1})}-\frac{{\left(P_{1}-P_{0}\right)}^{2}}{2}\frac{1}{2\pi Ta_{1}}\frac{da_{1}}{dP_{1}}. \end{array}$$

It has been taken into account in the above expression that

$$\frac{d}{dP_{1}}\left(\frac{1}{K(a_{1})}\right)=\frac{d}{dP_{1}}\left(\frac{\ln(R/a_{1})}{2\pi T}\right)=-\frac{1}{2\pi Ta_{1}}\frac{da_{1}}{dP_{1}}.$$

Hence, we get

4.7 $$\frac{dU_{T}}{dP_{1}}=(P_{1}-P_{0})\frac{d\delta_{1}}{dP_{1}}-\frac{\left(P_{1}-P_{0}\right)}{K(a_{1})}+\left[\frac{{\left(P_{1}-P_{0}\right)}^{2}}{4a_{1}\pi T}-2w\pi a_{1}\right]\left(\frac{da_{1}}{dP_{1}}\right)=0.$$

Using (3.17) of the above general statement, one obtains from (4.7) that the equilibrium condition for the general JKR model is

4.8 $$\frac{dU_{T}}{dP_{1}}=\left[\frac{{\left(P_{1}-P_{0}\right)}^{2}}{4a_{1}\pi T}-2w\pi a_{1}\right]\frac{da_{1}}{dP_{1}}=0$$

or

4.9 $${\left(P_{1}-P_{0}\right)}^{2}=8\pi^{2}wTa_{1}^{2}.$$

Further one has

$$P_{1}-P_{0}=\pi \sqrt{8wT}a_{1}=K(a_{1})\Delta \delta$$

and hence, the following expression is valid

$$\Delta \delta =\sqrt{\frac{2w}{T}}a_{1}\ln\left(\frac{R}{a_{1}}\right).$$

Thus, the general relations of the adhesive problem of contact between a graphene membrane and an arbitrary convex, smooth blunt axisymmetric punch *f*(*r*), *f*(0)=0, in the framework of the JKR approach are

4.10 $$P_{0}=P_{1}-\pi \sqrt{8wT}a_{1}\;\mathrm{and}\;\delta_{2}=\delta_{1}-\sqrt{\frac{2w}{T}}a_{1}\ln\left(\frac{R}{a_{1}}\right).$$

### (b) General expressions for power-law punches

Let us consider the problem for a power-law punches whose shape is described by (3.13). It follows from (3.15) that

4.11 $$P=2\pi mTB_{m}a^{m}.$$

Substituting (4.11) and (3.16) at *a*=*a*_1_ into (4.10), one get the expressions that solve the adhesive contact problem for an arbitrary power-law punch of degree *m*

4.12 $$P_{0}=2\pi T\left(mB_{m}a_{1}^{m-1}-\sqrt{\frac{2w}{T}}\right)a_{1}$$

and

4.13 $$\delta_{2}=mB_{m}a_{1}^{m}\left[\ln\left(\frac{R}{a_{1}}\right)+\frac{1}{m}\right]-\sqrt{\frac{2w}{T}}a_{1}\ln\left(\frac{R}{a_{1}}\right).$$

Evidently, the expressions (4.12) and (4.13) reduce to the above non-adhesive contact problems (the analogy to the Hertz-type contact problems) in the case *w*=0.

### (c) Tips of non-ideal shapes and dimensionless expressions

#### (i) Real shapes of indenter tips

Usually, for the experimental tests either special nanoindenters are used as the probe, or AFMs having an indentation testing function [7]. An important point in such studies is the geometric deviation of the probe shape from its nominal geometry, therefore, researchers employing nanoindenters developed various empirical area functions to relate the apparent contact area to depth. Borodich *et al.* [53] argued that at shallow depth, the nanoindenter blunt shapes are often well described by power-law functions of degree *m* with 1<*m*≤2. This point was later discussed by many researchers (for some references, see [44]). The same statement is applicable to actual shapes of probes used for contact mode AFM.

To describe the actual shapes of AFM tips, four different non-axisymmetric tips coated by diamond-like carbon were studied using both a special tip characterizer and the 2D images of the tip profiles obtained by scanning electron microscope. The values of *m* extracted from the power law approximations of the AFM tip geometry *z*=−*f*(*r*,*θ*)∼−*B*_*m*_(*r*,*θ*)*r*^*m*^ (*z*<30 nm) gave the values of *m* not close to 1 but rather *m*≈2. In addition, one has to realize that (i) nanoindenters drive the indenting tip always perpendicular to the surface during the depth-sensing nanoindentation test, while loading of a probe through an AFM cantilever involves the lateral movement and (ii) the AFM cantilever is mounted within an AFM device with a specific tilt angle (see a discussion in [54]). Hence, even if the AFM tip may be nominally rather sharp, the actual shape of a non-vertically fixed AFM tip near the first point of contact is rather blunt. Hence, the above expressions (4.12) and (4.13) can be used to describe the real adhesive contact between a probe tip and a membrane of 2D material.

#### (ii) Dimensionless expressions

An elastic half-space itself does not have any characteristic scale. Hence, there are two dilation similarity transformations of Hertz-type contact for a half-space [44,55]: (i) the punch shape function is transformed by the same homogeneous dilations along all axes and (ii) the punch shape function is transformed by dilation along the vertical axis only. The existence of these similarity transformations is the reason that the 3D Hertz-type contact problems are self-similar [56,57].

Owing to the absence of a characteristic scale, the choice of the characteristic parameters of a 3D adhesive contact problem is rather arbitrary [27]. For example, as the characteristic scale for a 3D adhesive contact problem, Borodich *et al.* [47] took the radius *a*_1_ of the contact region at *P*_0_=0. For 2D membranes, the dimensionless variables could be also specified using various characteristic scales, in particular, via the radius of the membrane. It follows from (4.12) that in the adhesive contact problem for a 2D material, the non-zero radius *a*_1_ of the contact region at *P*_0_=0 is

4.14 $$a_{1}(0)={\left(\frac{2w}{Tm^{2}B_{m}^{2}}\right)}^{1/2(m-1)},\;m>1.$$

One can see that *a*_1_(0) does not depend on the radius of the drum *R*. If this value is taken as a characteristic size of the contact region in order to write dimensionless parameters, then the characteristic parameters of the adhesive contact problems may be taken as

4.15 $$a^{*}=a_{1}(0),\;P^{*}={\left(\frac{\pi^{2(m-1)}2^{3m-2}w^{m}T^{m-2}}{m^{2}B_{m}^{2}}\right)}^{1/2(m-1)}\;\mathrm{and}\;\delta^{*}={\left(\frac{2^{m}w^{m}}{m^{2}T^{m}B_{m}^{2}}\right)}^{1/2(m-1)}.$$

In this case (4.12) and (4.13) have the following expressions

4.16 $$\frac{P_{0}}{P^{*}}={\left(\frac{a_{1}}{a^{*}}\right)}^{m}-\left(\frac{a_{1}}{a^{*}}\right)$$

and

4.17 $$\frac{\delta_{2}}{\delta^{*}}={\left(\frac{a_{1}}{a^{*}}\right)}^{m}\left[\frac{1}{m}-\ln\left(\frac{a_{1}/a^{*}}{R/a^{*}}\right)\right]+\frac{a_{1}}{a^{*}}\ln\left(\frac{a_{1}/a^{*}}{R/a^{*}}\right).$$

If we denote $\bar{P}=P_{0}/P^{*}$, $\bar{a}=a_{1}/a^{*}$ and $\bar{\delta }=\delta_{2}/\delta^{*}$, then (4.16) and (4.17) can be written as the following dimensionless relations

4.18 $$\bar{P}={\bar{a}}^{m}-\bar{a}$$

and

4.19 $$\bar{\delta }={\bar{a}}^{m}\left[\frac{1}{m}-\ln\left(\frac{\bar{a}}{\bar{R}}\right)\right]+\bar{a}\ln\left(\frac{\bar{a}}{\bar{R}}\right),\;\bar{R}=\frac{R}{a^{*}}$$

that are valid for an arbitrary axisymmetric monomial punch of degree *m*>1.

**Figure 3.**
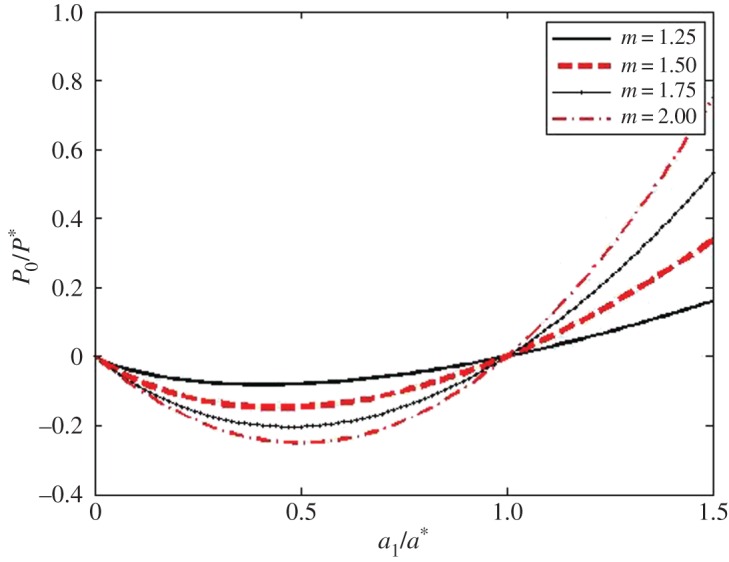
The dimensionless *P*_0_/P*–*a*_1_/a* relations (4.18) for monomial indenters of various degrees *m*. (Online version in colour.)

The above case of power-law shaped punches is important for probing membranes by indenters. Because the *P*_0_–*a*_1_ relation does not depend on the radii of the drum *R*, then in turn, the $\bar{P}$–$\bar{a}$ one does not also depend on *R*. The graphs of the dimensionless $\bar{P}$–$\bar{a}$ relations (4.21) for power-law indenters whose degree *m* are within the 0.25≤*m*≤2 range are shown in figure 3.

**Figure 4.**
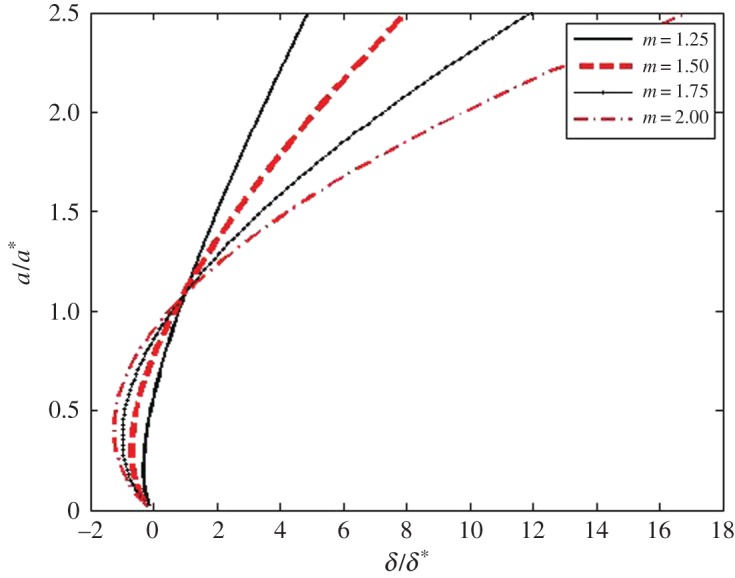
The dimensionless $\bar{a}$–$\bar{\delta }$ relations for power-law indenters for *m* within the 0.25≤*m*≤2 range. (Online version in colour.)

**Figure 5.**
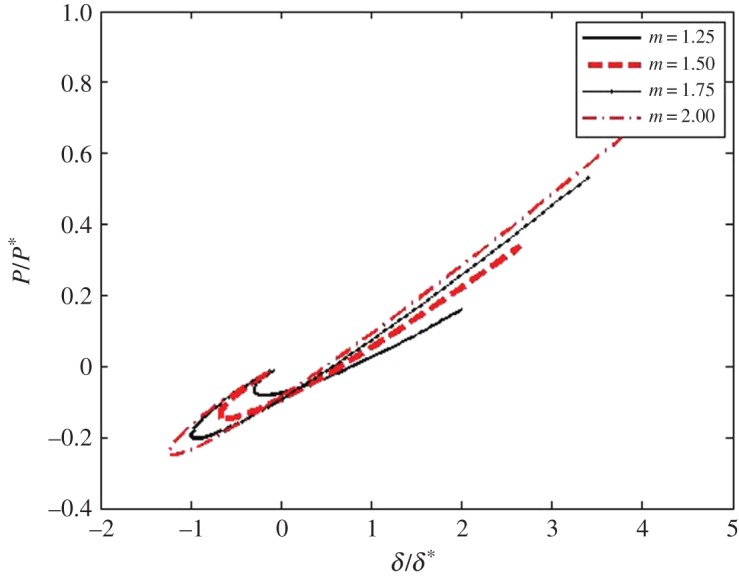
The dimensionless $\bar{P}$–$\bar{\delta }$ relations for power-law indenters for *m* within the 0.25≤*m*≤2 range. (Online version in colour.)

The graphs of the dimensionless $\bar{a}$–$\bar{\delta }$ relation for power-law indenters whose degree *m* are within the 0.25≤*m*≤2 range are shown in figure 4. Using the above $\bar{P}$–$\bar{a}$ and $\bar{a}$–$\bar{\delta }$ relations, one can get the graphs of the dimensionless $\bar{P}$–$\bar{\delta }$ relation for power-law indenters whose degree *m* is within the 0.25≤*m*≤2 range (figure 5). These and the above graphs have been obtained for $R/a=\bar{R}/\bar{a}=100$. One can see that in the case of blunt smooth punches, the general character of the new relations for 2D materials is similar to the character of the corresponding relations for elastic 3D materials [44,47,48].

Thus, the curve *P*–*δ* describes the depth-sensing indentation of the system under consideration. It is clear that if the external compressive load is not reduced then the indenter and the membrane jump out of the contact at the point d*P*/d*δ*=0, i.e. at the point of the tangent line is horizontal in experiments at fixed load *P* (e.g. [23,44]). This point can be calculated from the following relations

$$\frac{dP}{d\delta }=\frac{dP/da}{d\delta /da}=0\;\text{or}\;\frac{dP}{da}=0.$$

It follows from (4.18) that for *m*>1 the punch separates from the membrane at the critical radius ${\left(\bar{a}\right)}_{c}$

4.20 $${\left(\bar{a}\right)}_{c}=m^{1/(1-m)}$$

and at the corresponding critical load $\bar{P_{c}}$

4.21 $${\left(\bar{P}\right)}_{c}={\left(\bar{a}\right)}_{c}^{m}-{\left(\bar{a}\right)}_{c}=m^{m/(1-m)}-m^{1/(1-m)}=m^{1/(1-m)}(m^{-1}-1).$$

### (d) Spherical punches

The particular case of a spherical punch is very important for various applications. Let the punch be of radius *R*_*s*_, then *f*(*r*)=*B*_2_*r*^2^, *m*=2 and *B*_2_=1/(2*R*_*s*_).

For non-adhesive contact, one has

4.22 $$p(r)=\left\{ \begin{array}{ll} 2T/R_{s}=\mathrm{const}. & \text{for}\;r\leq a\\ 0 & \text{for}\;r>a. \end{array}\right.$$

It follows from (3.9) and (4.22) (or from (3.15)) that

4.23 $$\pi a^{2}\frac{2T}{R_{s}}=P\;\text{or}\;a=\sqrt{\frac{PR_{s}}{2\pi T}}$$

and one gets from (3.16) the following nonlinear expression for *δ*(*a*) under a sphere of radius *R*_*s*_

4.24 $$\delta =\frac{a^{2}}{2R_{s}}\left[2\ln\left(\frac{R}{a}\right)+1\right].$$

For adhesive contact, one can use the above general solution for monomial punches. It follows from (4.15): a*=*R*_*s*_(2*w*/*T*)^1/2^, P*=4*πR*_*s*_*w* and δ*=2*R*_*s*_*w*/*T*. Therefore, one obtains from (4.20) and (4.21):

$${\left(\bar{a}\right)}_{c}=\frac{1}{2}\;\text{and}\;{\left(\bar{P_{0}}\right)}_{c}=-\frac{1}{4}.$$

Thus, for a spherical punch, one gets explicit expressions for the values of the critical contact radius, displacement and the corresponding pull-off force in dimensional form

$$a_{c}=R_{s}\sqrt{\frac{w}{2T}},\;\delta_{c}=\frac{R_{s}w}{4T}\left[1+2\ln\left(\frac{R_{s}}{R}\sqrt{\frac{w}{2T}}\right)\right],\;\text{and}\;P_{c}=-\pi R_{s}w.$$

### (e) An analogy to the Boussinesq–Kendall adhesive contact problem

Consider an axisymmetric flat-ended punch of radius *a*_1_ that is vertically pressed into an elastic membrane. The elastic material deforms according to (3.26), i.e. *δ*=*P*_0_/*K*. The surface energy is given as above by (2.2). Using (3.26), one obtains that the stored elastic energy *U*_E_ and the mechanical energy of the applied load *U*_M_ are, respectively,

4.25 $$U_{E}=\int_{0}^{P_{0}}P\;d\delta =\frac{P_{0}^{2}}{2K}\;\mathrm{and}\;U_{M}=-P_{0}\delta =-\frac{P_{0}^{2}}{K}.$$

The total energy *U*_T_ can be obtained by summation of all components

4.26 $$U_{T}=-w\pi a_{1}^{2}-\frac{P^{2}}{2K}.$$

From the equilibrium equation (2.3), one has

4.27 $$\frac{dU_{T}}{da_{1}}=0=-2w\pi a_{1}+\frac{P_{c}^{2}}{4\pi Ta_{1}}$$

and, hence, one may obtain the adherence force (the pull-off force) of a flat-ended circular punch of radius *a*_1_

4.28 $$P_{c}=2\pi a_{1}\sqrt{2wT}.$$

Studying the frictionless Boussinesq–Kendall adhesive contact problem for an elastic-half space, Maugis [23] came to the conclusion that the adherence force is proportional neither to the energy of adhesion nor to the area of the contact. One can see from (4.28) that the same conclusion is valid for a circular elastic membrane, but the adherence force is proportional to the perimeter of the punch.

## 5. Conclusion

It has been argued that to study mechanical properties of atomically thin materials such as graphene or graphene oxide, their discrete structure may be modelled as a stretched elastic membrane whose bending rigidity is neglected. For such membranes, we have formulated and solved axisymmetric contact problems that are analogous to the Hertz and Boussinesq frictionless contact problems. For these new problems, a general statement for the slope of the force–displacement curve has been formulated and proved. The results may be used not only in application to monoatomic thick 2D materials but also to membranes of other types, e.g. few-layer graphene sheets [2], the graphene oxide paper [58] and other thin films.

Because attractive surface forces may be very significant at nanometer scale, analogies to the JKR and the Boussinesq–Kendall contact problems in the presence of adhesive interactions have been formulated. General nonlinear equations are derived for the relations between the actual external force, the probe displacements and the contact radius in the case of an arbitrary smooth, convex axisymmetric indenter. Note that the pressure distribution under action of a rigid punch has the delta-function singularity on the circular line of the contact radius *a*_1_.

Note that we have studied the problems applicable to the real tips of both nanoindenter and AFM probes that may be normally described by power law functions of degrees *m*>1. We have excluded from our consideration the adhesive contact problem for a cone (*m*=1). This case will be studied in another publication. For power-law shaped axisymmetric indenters, the dimensionless form of the equations has been analysed, and the explicit expressions are derived for the values of the pull-off force and for the corresponding critical contact radius. The particular cases of spherical indenters and flat-ended circular indenters have been considered in detail. The approach presented may be a ground for development new techniques for experimental studies of mechanical properties of 2D materials.

As it has been noted by an anonymous reviewer, considering that all of the problems discussed have radial symmetry, equations (3.5) and (3.6) could have been developed without reference to any Green’s function. Equally, the jump condition across *r*=*a* could have been given directly, and all of the solutions (non-adhesive and adhesive) could have been derived directly from the ordinary differential equation and the boundary and jump conditions. We agree with this comment, however, we would like to add that we intend to use the Green’s function approach in our future papers for solving non-axisymmetric contact problems.

## Data accessibility

All of the data is contained within this article.
